# Effects of mGluR5 Antagonists on Parkinson's Patients With L-Dopa-Induced Dyskinesia: A Systematic Review and Meta-Analysis of Randomized Controlled Trials

**DOI:** 10.3389/fnagi.2018.00262

**Published:** 2018-09-11

**Authors:** Wen-Wen Wang, Xing-Ru Zhang, Zeng-Rui Zhang, Xin-Shi Wang, Jie Chen, Si-Yan Chen, Cheng-Long Xie

**Affiliations:** ^1^The Center of Traditional Chinese Medicine, The Second Affiliated Hospital and Yuying Children's Hospital of Wenzhou Medical University, Wenzhou, China; ^2^Department of Neurology, The First Affiliated Hospital of Wenzhou Medical University, Wenzhou, China

**Keywords:** mGluR5 antagonists, parkinson's disease, L-dopa-induced dyskinesia, meta-analysis, systematic review

## Abstract

**Background:** Modulation of Metabotropic glutamate receptor 5 (mGluR5) may be a novel therapeutic approach to manage Parkinson's disease (PD) Patients with L-dopa-induced dyskinesia (LID).

**Objectives:** The objective of this meta-analysis was to evaluate the effects of mGluR5 antagonists for the treatment of LID patients.

**Methods:** Several electronic databases were consulted up to July 30, 2017. Randomized clinical trials (RCTs) that compared mGluR5 antagonists vs. placebo in LID patients were included. Pooled weighted mean difference (WMD) with 95% confidence intervals (CIs) were calculated using random-effects models.

**Results:** Nine trials including 776 patients met all inclusion criteria. We pooled the whole data and found apparent difference between mGluR5 antagonists and placebo in terms of mAIMS (*p* = 0.010). However, there was no significant improvements on antidyskinetic in terms of LFADLDS (*p* = 0.42) and UPDRS Part IV (*p* = 0.20). Meanwhile, the effect size of UPDRS part III was similar in mGluR5 antagonist groups with in placebo groups (*p* = 0.25). Adverse events incidence was higher with mGluR5 antagonists than with placebo, especially at the expense of increased dizziness (16.3 vs. 4.3%), visual hallucination (10.1 vs. 1.1%), or fatigue (10.1 vs. 4.8%).

**Conclusions:** mGluR5 antagonists had a greater treatment effect on the mAIMS in LID patients, however, there was no improvements on antidyskinetic in terms of LFADLDS and UPDRS Part IV compared with placebo. According to these results, we unable to recommend mGluR5 antagonists for the routine treatment of LID patients right now.

## Introduction

Dopamine-replacement therapy using L-dopa (L-3,4-dihydroxyphenylalanine) remains the most effective drug for treating Parkinson's disease (PD). However, long-term use of L-dopa is associated with L-dopa-induced dyskinesia (LID), which can become treatment-limiting (Jenner, 2008). More than 50% of PD patients will develop LID between 5 and 10 years after beginning L-dopa therapy (Calabresi et al., 2010). Until now, the potential neuropathological mechanisms of LID were largely unknown (Bastide et al., 2015). There are limited options for managing LID without deteriorating parkinsonism, such as fine adjustment of L-dopa doses, amantadine, prolonged dopaminergic stimulation, and deep brain stimulation (DBS) (Fox et al., 2011).

Among these options, amantadine, a weak antagonist of the ionotropic N-methyl-D-aspartate (NMDA) receptor, is the sole pharmacological agent used to treat LID patients in the clinic, indicating that increased glutamatergic neurotransmission is associated with the pathophysiology of LID (Del-Bel et al., 2016). The metabotropic glutamate receptors (mGluR), are a type of glutamate receptor that are active through an indirect metabotropic process (including mGluR1-8). They are members of the group C family of G-protein-coupled receptors (Bonsi et al., 2005). The mGluR perform a variety of functions in the central and peripheral nervous systems. For example, they are involved in learning, memory, anxiety, and the perception of pain (Reiner and Levitz, 2018). Mohamed et al. reported that for development of melanoma, activation of every signaling pathway from mGluR1 is required (Abdel-Daim et al., 2010). In addition, inactivation of the mGluR1 transgene in melanoma mice inhibited melanoma growth with reduction of phosphorylated ERK1/2, whereas mice with abiding expression of mGluR1 developed larger melanoma burdens (Ohtani et al., 2008). Moreover, in terms of LID field, a previous study in non-human primates reported that LID was related to increased mGluR5-specific binding in the striatum vs. controls (Samadi et al., 2008). Similarly, LID patients have higher mGluR5-specific binding in the basal ganglia than patients without dyskinesia (Ouattara et al., 2011). Accordingly, mGluR5 antagonists have been studied as a potential treatment for reducing LID symptoms in PD patients. Nevertheless, trials investigating the effectiveness of mGluR5 antagonists have yielded mixed results, possibly due to low statistical power and extensive variation in treatment regimens (Chou et al., 2015). Rajeev et al. demonstrated that mavoglurant, which is a subtype-selective and noncompetitive antagonist of the mGluR5 binding site, combined with higher doses of L-dopa may be effective in treating patients with PD experiencing L-dopa-related motor fluctuations and dyskinesia (Kumar et al., 2016). On the other hand, Francois et al. reported that dipraglurant, a novel mGluR5 antagonist, was safe and tolerable. Its efficacy in reversing LID warrants further investigations in more patients (Tison et al., 2016).

Hence, it is unclear whether mGluR5 antagonists are more effective in LID patients due to the inconsistent results. It is critical to integrate and arrange these findings to accurately determine the effects of mGluR5 antagonists on LID. Therefore, the aim of this study was to conduct a direct meta-analysis of standard treatments in LID patients from randomized clinical trials (RCTs) to compare mGluR5 antagonists vs. placebos.

## Materials and methods

### Search strategy

To identify relevant studies for inclusion in this meta-analysis, we searched PubMed, Google Scholar, and the Cochrane Library from inception through July 30, 2017. To avoid omitting relevant trials, we also searched conference summaries and reference lists from general reviews on mGluR5 treatment in advanced PD. Two reviewers (XRZ and ZRZ) independently screened the titles, abstracts, and references from all identified reports.

The Medline (PubMed) search strategy was as follows:

#1. (Parkinson's disease [mh]) OR (Idiopathic Parkinson's Disease) OR (Lewy Body Parkinson Disease) OR (Lewy Body Parkinson's Disease) OR (Primary Parkinsonism) OR (Parkinsonism, Primary) OR (Paralysis Agitans)
#2. (Metabotropic glutamate receptor 5 [mh]) OR (mGluR5) OR (mGluR5b Protein) OR (Metabotropic Glutamate Receptor 5b) OR (Metabotropic Glutamate Receptor 5a) OR (Protein, mGluR5) OR (Lithium) OR (LY-344,545) OR (Mavoglurant) OR (Remeglurant) OR (SIB-1893)
#3. (dyskinesia [mh]) OR (L-dopa-induced dyskinesia) OR (LID)
#4. #1 OR #2 OR #3

### Inclusion and exclusion criteria

We included clinical trials that met the following criteria: (1) randomized controlled design trials comparing mGluR5 antagonists with placebo to treat idiopathic advanced PD, (2) trials with participants aged 30 years or older and masked assessment of outcomes, (3) trials describing advanced PD patients with L-dopa-induced dyskinesia, and (4) reports that were published in English. mGluR5 antagonists are defined as a drug with reported antagonism on mGluR5 irrespective of actions at other receptors. Trials were excluded for the following reasons: (1) trials that were not RCTs, including case reports, abstracts, comments, reviews, and editorials; (2) trials conducted in non-human subjects; (3) trials that did not conduct tests to evaluate the effects of mGluR5 antagonists on LID patients; and (4) reports that were duplicate publications.

### Data extraction

For each trial, detailed information was carefully extracted from all the eligible trials, including the first author, year of publication, sample size, and basic sample characteristics, study population, study design, mGluR5 antagonist protocols, and outcome measures. Outcome measures included modified Abnormal Involuntary Movements Scale (mAIMS) (Marconi et al., 1994), Lang-Fahn Activities of Daily Living Dyskinesia Scale (LFADLDS) (Parkinson Study Group, 2001), Unified Parkinson's Disease Rating Scale (UPDRS), and adverse events. Finally, we also extracted the main results of the included trials.

### Risk of bias

The risk of bias for included trials was assessed independently using the Cochrane Handbook for Systematic Reviews of Interventions (Higgins et al., 2011). The details of risk of bias analysis are consistent with our previous paper (Xie et al., 2016).

### Statistical analysis

All statistical analyses were done in accordance with the intention-to-treat (ITT) principle. In this study, primary outcome measures (such as LFADLDS, mAIMS and UPDRS) were treated as continuous data. Weighted mean differences (WMD), standard statistics that measure the absolute difference between the mean values in two groups, were given in this meta-analysis. The mean effect was expressed as WMD with 95% confidence intervals (Vesterinen et al., 2014). We adapted a random effect model rather than a fixed effect model to measure pooled effect sizes. We chose the random model because it takes into account the potential clinical or methodologic heterogeneity between multiple studies and also yields a more conservative estimate of the pooled effect. We used the Q statistic and the *I*^2^ index to assess the statistical heterogeneity. A probability value of *P* ≤ 0.1 and an *I*^2^ value >50% are indicative of heterogeneity between included studies, as the values exceed what is expected by chance (Higgins et al., 2003). If outcomes were presented at different time points, we extracted data from the last time point of analysis. If means and standard deviations were not provided, we calculated them from standard errors, CI, or other statistical indices. We performed a sensitivity analysis to examine whether our results would have differed by removing each individual study from the total and reanalyzing the remainder. Finally, we used the Grading of Recommendations, Assessment, Development and Evaluation (GRADE) approach to assess the quality of evidence. There are four levels for rating quality of evidence: high, moderate, low, and very low (Atkins et al., 2004). Publication bias was not assessed due to the limited trials. All analyses were performed with RevMan version 5.1. Probability value of *P* < 0.05 was considered significant.

## Results

### Results of the search

**Figure 1:**
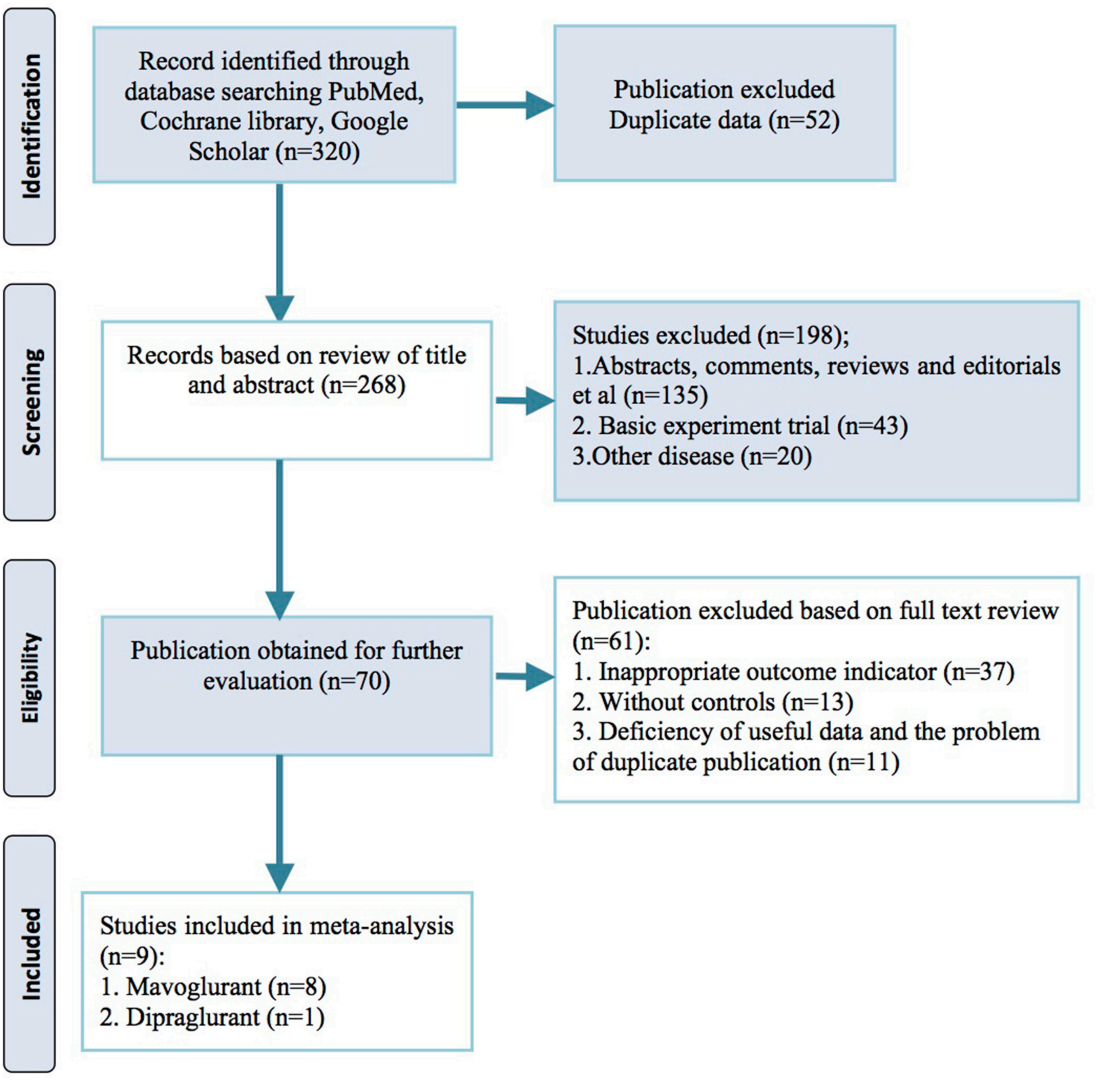
PRISMA 2009 flow diagram.

Our initial search of electronic databases yielded 320 publications. After screening through titles, abstracts and full texts, 9 trial reports were identified for the final meta-analysis (Berg et al., 2011; Stocchi et al., 2013, 2014; Trenkwalder et al., 2014, 2016; Kumar et al., 2016; Tison et al., 2016). No further studies were identified by manual searches of the three journals from which most eligible studies were identified electronically (*Movement Disorders, Parkinsonism & Related Disorders* and *Neurology*). Of the resulting 9 reports that were reviewed in full text, 8 studies used mavoglurant as the mGluR5 antagonist and the 1 remaining study used dipraglurant (Figure 1).

### Study characteristics

Nine RCTs, with a total of 776 LID patients, met the inclusion criteria and were included in this review. Among the patients, 507 were randomized to mGluR5 antagonist groups, and 269 were randomized to placebo groups. The number of subjects included in this meta-analysis ranged from 14 to 197. Meanwhile, the time of follow-up ranged from 16 days to 12 weeks. The mGluR5 antagonists used in this study include mavoglurant (*n* = 8) and dipraglurant (*n* = 1). For outcome measures, LFADLDS was recorded in 6 trials, mAIMS in 8 trials, and UPDRS Parts III and IV in 7 and 6 trials, respectively. One paper by Daniela et al. (Berg et al., 2011) contained two trials (NCT00582673 and NCT00888004); similarly, one paper by Claudia et al. (Trenkwalder et al., 2016) included two phase 2 randomized, double-blind trials (NCT01385592 and NCT01491529). Two trials have not yet been published (Higgins et al., 2003; Atkins et al., 2004), and the information of two other trials with mavoglurant are due to be presented at the 18th International Congress of Parkinson's disease and Movement Disorders. The basic characteristics of the 9 trials are summarized in Table 1.

**Table 1:** Basic information of the included trials.

**Study (years)**	**Basic data**				
	**Study drug**	**Age (EG; CG) (Mean ± *SD*)**	**Male/Female (EG;CG)**	**Study population**	**Sample size**	**Method**	**Outcome indicators**	**Results**
Berg et al., 2011	Mavoglurant	60.7 ± 10.58; 61.4 ± 10.28	9/6;7/9	Moderate-to-severe LID	31 (Mavoglurant, *n* = 15; placebo, *n* = 16)	Mavoglurant 25–150 mg or placebo bid for 16 days	1. LFADLDS 2. UPDRS-III 3. mAIMS 4. UPDRS-IV items 32–33	1. Mavoglurant showed significant antidyskinetic effects vs. placebo on the LFADLDS, mAIMS and UPDRS-IV on day 16. 2. No significant difference between Mavoglurant and placebo on the UPDRS-III on day 16. 3. Most common AE: dizziness, not dose-related.
	Mavoglurant	65.6 ± 7.54; 66.1 ± 6.55	9/5;8/6	Severe LID	28 (Mavoglurant, *n* = 14; placebo, *n* = 14)	Mavoglurant 25–150 mg or placebo bid for 16 days; 4-day down-titration (50 mg Mavoglurant bid)	1. mAIMS 2. UPDRS-III 3. LFADLDS 4. UPDRS-IV items 32-33	1. Mavoglurant showed significant antidyskinetic effects vs. placebo on the mAIMS on day 16 (*P* = 0.32). 2. No significant difference between Mavoglurant and placebo on the UPDRS-III on day 16. 3. Significant treatment effects were seen on the UPDRS-IV on days 12, 16, 20 (*P* = 0.026, *P* = 0.001 and *P* = 0.077 vs. placebo, respectively). 4. Trend toward improvement on LFADLDS. 5. Most common AE: dizziness; not dose-related.
Stocchi et al., 2013	Mavoglurant	66.2 ± 8.16 (EG20); 66.4 ± 7.96 (EG50); 65.6 ± 9.47 (EG100); 66.0 ± 10.54 (EG150); 63.4 ± 8.98 (EG200); 64.8 ± 8.17	10/12 (EG20); 13/9 (EG50); 14/9 (EG100); 14/8 (EG150); 24/20 (EG200); 30/34	Moderate-to-severe LID	197 (Mavoglurant, *n* = 133; placebo, *n* = 64)	Receiving either Mavoglurant (at doses of 20, 50, 100, 150, or 200 mg daily) or placebo (1:1:1:1:2:3 ratio) for 12 weeks	1. mAIMS 2. PDYS-26	1. Mavoglurant 200 mg daily group differ significantly from placebo on the mAIMS (*P =* 0.007 vs. placebo). 2. No significant treatment effects were observed in PDYS-26 scores for any Mavoglurant group versus placebo. 3. Most common AE: dizziness, hallucination, fatigue, nasopharyngitis, diarrhea, and insomnia.
Stocchi et al., 2014	Mavoglurant	NR	NR	Moderate-to-severe LID	61 randomized to Mavoglurant or placebo (4:3)	Receiving Mavoglurant 100 mg bid immediate release or placebo for 12 weeks; Titration of Mavoglurant was at 2 weeks intervals	1. mAIMS 2. LFADLDS 3. CGIC 4. UPDRS-III 5. UPDRS-IV 6. patient diary	1. The treatment difference between the Mavoglurant100 mg and placebo groups was not significant (−1.7 ± 1.31; *p* = 0.2095) for mAIMS. 2. No significant changes were observed on any of the secondary measures. 3. Most common AE: dizziness and visual hallucination.
Trenkwalder et al., 2014	Mavoglurant	NR	NR	Moderate-to-severe LID	154 patients were randomly assigned to Mavoglurant or placebo (2:1)	Receiving Mavoglurant (target dose of 150 or 200 mg bid modified release) or placebo; Titration of Mavoglurant was at 2-week intervals	1. mAIMS 2. LFADLDS 3. UDysRS 4. CGIC 5. UPDRS-III 6. UPDRS-IV 7. patient diary	1. No significant change was observed on mAIMS in the 200 mg bid (−0.2 ± 1.03; *p* = 0.8640 vs. placebo) or 150 mg (−1.3 ± 1.16; *p* = 0.2707 vs. placebo) groups. 2. No significant differences were observed on any secondary measure. 3. Most common AE:visual hallucination.
Kumar et al., 2016	Mavoglurant	61.3 ± 8.98; 61.4 ± 6.00	2/5;4/3	Moderate-to-severe LID	14 (Mavoglurant, *n* = 7; placebo, *n* = 7)	Screening(4 weeks); Mavoglurant/placebo up-titration (2 weeks); L-dopa up-titration(3 weeks); Blinded taper-off (1 week); Follow-up (1 week)	1. total OFF-time 2. total ON-time	1. Patients in the mavoglurant group experienced a clinically meaningful improvement in OFF-time at week 5 vs. placebo. 2. Patients in the mavoglurant group also showed a greater improvement in ON-time without troublesome dyskinesia at week 5(4.38 h) compared with the placebo group (0.63 h). 3. Most common AE: dyskinesia.
Tison et al., 2016	Dipraglurant	64.2 ± 7.6; 62.8 ± 8.3	26/26;12/12	Moderate-to-severe LID	76 (Dipraglurant, *n* = 52; placebo, *n* = 24)	Dipraglurant(dose titration from 50 mg qd on day 1 to 50 mg tid days 7–13 and up to 100 mg tid days 14–21) or placebo for 4 weeks	1. Safety and Tolerability 2. mAIMS 3. UPDRS-III 4. patient diary 5. UPDRS 6. CGIC	1. Dipraglurant has significant efficacy on mAIMS at day 1 (*p* = 0.042) and day 14 (*P* = 0.034); mAIMS (AUC 0-3) at day 14 (*P* = 0.042) 2. UPDRS Part III: Dipraglurant did not affect levodopa efficacy. 3. Dipraglurant increased daily On time and reduced daily Off time. 4. CGIC dyskinesia showed greater improvement in dyskinesia for dipraglurant. 5. No significant changes in safety monitoring parameters. 6. Most common AE:dyskinesias,dizziness and nausea.
Trenkwalder et al., 2016	Mavoglurant	65.9 ± 6.97; 66.6 ± 7.04	19/17;15/10	Moderate-to-severe LID	61(Mavoglurant, *n* = 36; placebo, *n* = 25)	4 weeks screening period; 2 weeks single-blind placebo run-in period; 12 weeks double-blind treatment: the mavoglurant dose was gradually up-titrated every 2 weeks until the target dose was reached	1. mAIMS 2. LFADLDS 3. UPDRS-III 4. UPDRS-IV 5. CGIC 6. patient diary	1. The difference in mAIMS total score was not statistically significant (mavoglurant 100 mg vs. placebo; *P* = 0.2095). 2. There was no significant difference between the mavoglurant 100-mg and placebo groups for any of the other secondary efficacy outcomes.
	Mavoglurant	64.4 ± 8.68 (EG150); 64.4 ± 8.84 (EG200); 64.2 ± 9.02	22/17 (EG150); 41/37 (EG200); 21/16	Moderate-to-severe LID	154 patients were randomly assigned to mavoglurant 200- or 150 mg or placebo (2:1:1)	4 weeks screening period; 2 weeks single-blind placebo run-in period; 12 weeks double-blind treatment; 1 week, randomized, blinded taper-off period(mavoglurant 50 mg bid. for 3 days followed by 25 mg bid. for 4 days)	1. mAIMS 2. LFADLDS 3. UPDRS-III 4. UPDRS-IV 5. CGIC 6. patient diary 7. UDysRS	1. No statistical significance was observed for the treatment difference in mAIMS total score between mavoglurant group and the placebo group in week 12. 2. In individual groups there was no significant difference in the mAIMS mean change from baseline in week 12. 3. There was no significant difference between the mavoglurant 100 mg and placebo groups for any of the other secondary efficacy outcomes.

*EG, experimental group; CG, control group; SD, standard deviation; mAIMS, modified Abnormal Involuntary Movements Scale; CGIC, the Clinician-rated Global Impression of Change; LFADLDS, Lang-Fahn Activities of Daily Living Dyskinesia Scale; UPDRS, Unified Parkinson's Disease Rating Scale; UDysRS, Unified Dyskinesia Rating Scale; PDYS-26, 26-item PD Dyskinesia Scale*.

### Risk of bias

**Figure 2:**
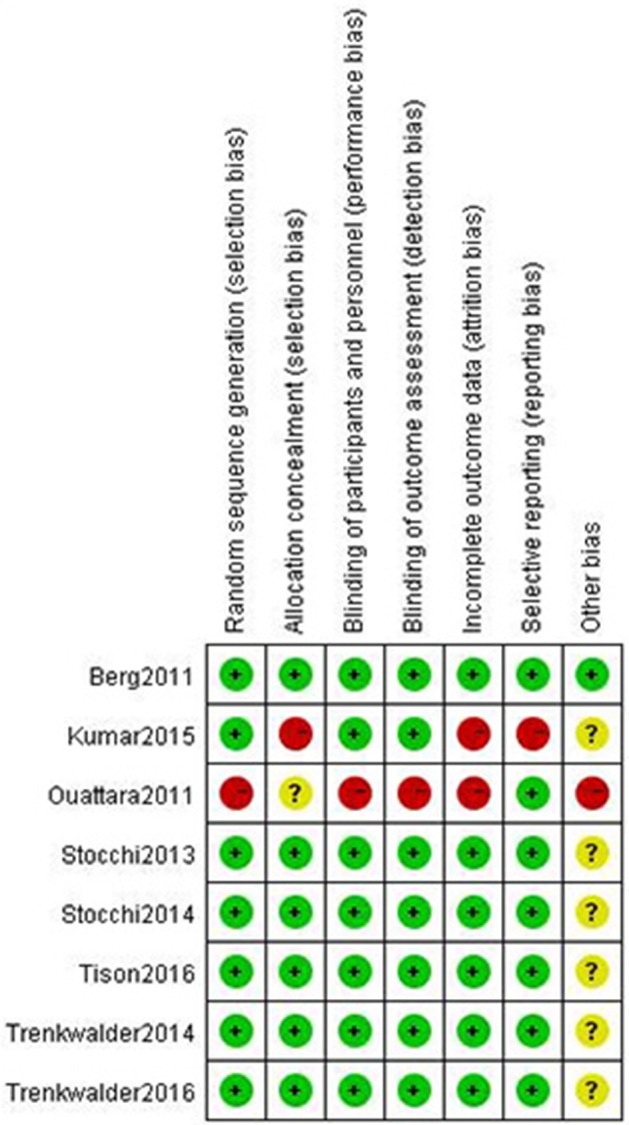
Risk of bias of included trials using the Cochrane Handbook for Systematic Reviews of Interventions.

Figure 2 shows the risk of bias in the included trials. Eight trials described the method of randomization used. Seven trials assessed whether an adequate concealment of allocation procedure was used, and eight trials reported methods for blinding participants. Seven trials described intention-to-treat analyses (ITT) and reported follow-up data. Selective reporting was found in one trial. Therefore, all of the included trials were determined to have a low risk of bias.

### Meta-analyses

**Figure 3:**
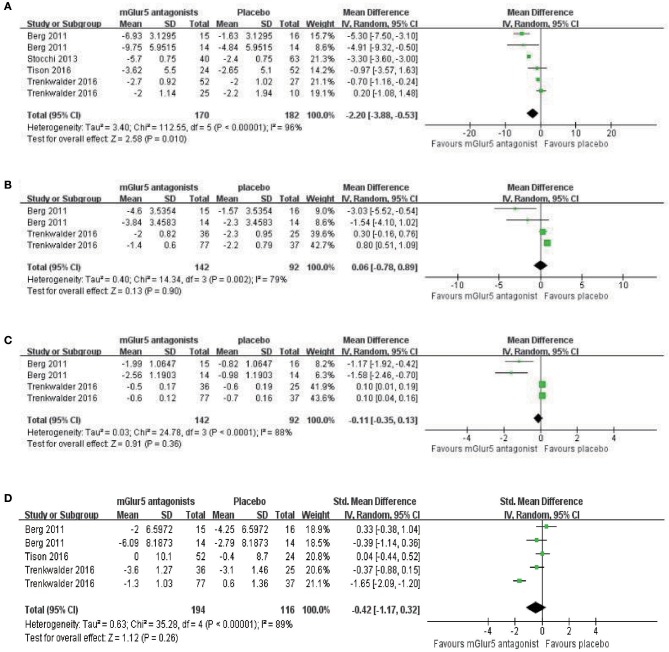
Forest plot of effect sizes for **(A)** mAIMS, **(B)** LFADLDS, **(C)** UPDRS Part IV, and **(D)** UPDRS part III: Mean changes from baseline to end point. mAIMS, modified Abnormal Involuntary Movements Scale LFADLDS, Lang-Fahn Activities of Daily Living Dyskinesia Scale; UPDRS, Unified Parkinson's Disease Rating Scale.

**Antidyskinetic:** In this meta-analysis, mAIMS was available from 6 trials of mGluR5 antagonists compared with placebo and showed a significant difference between the two groups (*p* = 0.010, WMD = −2.20, 95% CI: −3.88 to −0.53, Figure 3A). Meanwhile, there was obvious heterogeneity in the analysis of mAIMS between trials (Chi^2^ = 112.55, *p* < 0.00001, *I*^2^ = 96%, Figure 3A). Two trials failed to pool analysis due to the data being deficient, and neither reported significant differences observed between the two groups (*p* = 0.2095 and *p* = 0.8640, respectively). For LFADLDS, no significant difference was obtained between mGluR5 antagonists and placebo based on four trials (*n* = 234, WMD = 0.06, 95% CI: −0.78 to 0.89, *P* = 0.90; heterogeneity: Chi^2^ = 14.34, *p* = 0.002, *I*^2^ = 79%, Figure 3B). The remaining two trials were not included in the meta-analysis due to the lack of detailed data reported. Analogously, neither of them reported significant effects of mavoglurant in reducing LFADLDS scores compared with placebo group (*p* > 0.05). Moreover, we merged all the data and discovered no significant difference between mGluR5 antagonists and placebo according to UPDRS Part IV (*n* = 234, WMD = −0.11, 95% CI: −0.35 to 0.13, *P* = 0.36; heterogeneity: Chi^2^ = 24.78, *p* < 0.0001, *I*^2^ = 88%, Figure 3C). **Antiparkinsonian: Four** trials reported no significant effect of mGluR5 antagonists for reducing UPDRS Part III scores compared with placebo (*n* = 310, *p* = 0.26, WMD = −0.42, 95% CI: −1.17 to 0.32, Figure 3D). Meanwhile, all the results showed heterogeneity when using the heterogeneity test (Chi^2^ = 35.28, *p* < 0.00001, *I*^2^ = 89%, Figure 3D).

### Adverse events

In the nine studies, the incidence of AEs was higher in patients in the mGluR5 antagonist groups than in the placebo groups (Table 2). AEs were typically mild to moderate in severity and included nervous system, psychiatric, and gastrointestinal disorders. Among them, the most common AEs were dizziness (16.3% in mGluR5 antagonist groups vs. 4.3% in placebo groups), visual hallucination (10.1 vs. 1.1%), fatigue (10.1 vs. 4.8%), insomnia (6.1 vs. 1.6%), nasopharyngitis (6.1 vs. 1.6%) and diarrhea (5.1 vs. 2.1%). Overall, 10.1% of patients were reported to have dyskinesia in the mGluR5 antagonist groups, which was lower than 21.9% in the placebo groups. No deaths were reported in either of the treatment groups in any of the trials.

**Table 2:** Summary of most common adverse events.

**Adverse events**	**mGluR5 antagonists *N* = 374**	**Dipraglurant *N* = 52**	**Mavoglurant *N* = 322**	**Placebo *N* = 187**
**MOST COMMON AEs^a^, *n* (%)**				
Dizziness	61(16.3)	–	61 (18.9)	8 (4.3)
Dyskinesia	38 (10.1)	–	38 (11.8)	41 (21.9)
Visual hallucination	38 (10.1)	–	38 (11.8)	2 (1.1)
Fatigue	38 (10.1)	8 (15.4)	30 (9.3)	9 (4.8)
Insomnia	23 (6.1)	–	23 (7.1)	3 (1.6)
Nasopharyngitis	23 (6.1)	–	23 (7.1)	3 (1.6)
Diarrhea	19 (5.1)	–	19 (5.9)	4 (2.1)
Confusional state	16 (4.3)	–	16 (5.0)	1 (0.5)
Illusion	16 (4.3)	–	16 (5.0)	1 (0.5)
Nausea	16 (4.3)	–	16 (5.0)	8 (4.3)
Fall	15 (4.1)	3 (5.8)	12 (3.7)	4 (2.1)
Headache	16 (4.3)	6 (11.5)	10 (3.1)	7 (3.7)
Hypertension	7 (1.9)	5 (9.6)	2 (0.6)	1 (0.5)
Asthenia	5 (1.3)	4 (7.7)	1 (0.3)	4 (2.1)
On and off phenomena	6 (1.6)	6 (11.5)	–	2 (1.1)
Vertigo	4 (1.1)	4 (7.7)	–	0
Visual impairment	4 (1.1)	4 (7.7)	–	0
Feeling drunk	3 (0.8)	3 (5.8)	–	0
Somnolence	3 (0.8)	3 (5.8)	–	3 (1.6)
Others AEs, n (%)	114 (30.5)	–	114 (35.4)	29 (15.5)

a *These were events that were reported by at least 4% of dipraglurant-treated or mavoglurant-treated patients; AE, adverse event*.

### Quality of the meta-analysis

Using the GRADE criteria, we characterized the quality of evidence presented in this meta-analysis as low to moderate. LFADLDS and UPDRS Part IV had a low level of evidence across all domains, and mAIMS and UPDRS Part III had a moderate level of evidence. The basic information for all core comparisons of the outcomes of the included trials is summarized in Table 3.

**Table 3:** Risk of bias assessment across studies by GRADE criteria.

**Outcome measure**	**LFADLDS**	**mAIMS**	**UPDRS-III**	**UPDRS-IV items 32–33**
N of studies	4	6	5	4
**DOWNGRADE QUALITY OF EVIDENCE**				
Risk of bias	No	No	No	No
Inconsistency	Serious	Serious	No	Serious
Indirectness	Serious	Serious	Serious	Serious
Imprecision	No	No	No	No
Publication bias	Undetected	Undetected	Undetected	Undetected
**UPGRADE QUALITY OF EVIDENCE**				
Large effect	No	No	No	No
Plausible confounding would change the effect	No	No	No	No
Dose-response gradi	No	Yes	No	No
Effect (95%CI)	0.06 (−0.78 to 0.89)	−2.2 (−3.88 to −0.53)	−0.42 (−1.17 to 0.32)	−0.11 (−0.35 to 0.13)
Quality of evidence	Low	Moderate	Moderate	Low

*LFADLDS, Lang-Fahn Activities of Daily Living Dyskinesia Scale; mAIMS, modified Abnormal Involuntary Movements Scale; UPDRS, Unified Parkinson's Disease Rating Scale*.

## Discussion

### Summary of main results

Several conclusions can been draw based on the findings of this meta-analysis. First, our meta-analysis found that mGluR5 antagonists had a greater treatment effect on mAIMS scores (*p* = 0.01) in LID patients. However, there were no significant improvements in anti-dyskinesia in LFADLDS (*p* = 0.42) and UPDRS Part IV (*p* = 0.20) compared with placebo, as well as no effects on anti-parkinsonism (*p* = 0.25). Second, 9 trials evaluated the incidence of adverse events and none were reported. There were no major safety concerns associated with mavoglurant (100–200 mg) or dipraglurant (50–300 mg). The incidence of AEs was higher in patients in the mGluR5 antagonist groups than in the placebo group, especially increased dizziness, visual hallucination and fatigue. Finally, using the GRADE system, we characterized the quality of evidence in our meta-analysis as low to moderate. To our best knowledge, the meta-analysis was unable to determine if mGluR5 antagonists were beneficial in the treatment of PD or LID patients.

### Interpretation of the results

The development of LID is associated with increased glutamatergic signaling in the basal ganglia, particularly in its major input system (Chase and Oh, 2000). Inhibition of glutamatergic transmission signals by the selective mGluR5 antagonists have been shown to alleviate LID in rodent and monkey models of PD (Levandis et al., 2008; Morin et al., 2010). Moreover, the NMDA-receptor antagonist amantadine has shown effectiveness in ameliorating LID in patients, although its transitory effects and multiple neurological side effects in some patients limit its clinical use (Bibbiani et al., 2005). Consistently, our meta-analysis found that mGluR5 antagonists had a greater treatment effect compared with placebo on mAIMS scores in LID patients. Nevertheless, based on the combined analysis, we did not find other secondary end points with consistent improvements in dyskinesia scores (LFADLDS and UPDRS Part IV) for mGluR5 antagonists compared to placebo. Namely, the results on clinician-rated measures of dyskinesia were conflicting; a slight improvement was observed in the mAIMS scores, while the LFADLDS and UPDRS Part IV scores were not different between mGluR5 antagonists and placebo. The reasons for this discrepancy in the study outcomes were not apparent, but there were differences in the design of these trials and the trial sample was limited. Meanwhile, the LFADLDS and UPDRS Part IV scales may not have been sensitive enough to detect the observed antidyskinetic effects, or the magnitude of therapeutic effect may be too small to generate a change on these measures (Goetz et al., 2013). Therefore, these results should be interpreted with caution and additional large multi-center RCTs are required to assess the full potential of mGluR5 antagonists in patients with LID. Moreover, mGluR5 antagonists were not related to any significant effects on the UPDRS Part III scores in either trial, suggesting mGluR5 antagonists did not alter the antiparkinsonian effect of L-dopa therapy.

### Limitations

Several limitations of this meta-analysis should be mentioned. First, a relatively small sample size of LID patients was included from seven papers with nine trials. Our results could be constrained by the unclear risk of bias due to incomplete data in a few trials. Second, the longest duration of treatment was 12 weeks; therefore, the long-term antidyskinetic effects and cost-effectiveness of mGluR5 antagonists could not be assessed here. Additionally, a few uncontrolled variables, such as disease stage, medication use, and types of mGluR5 antagonists, could confound the results. Third, mGluR5 antagonists in this study include only mavoglurant (*n* = 8) and dipraglurant (*n* = 1) because the other antagonists trials were not identical and may have influenced our results. Moreover, only one trial used dipraglurant. Finally, two trials have not yet been published, but will be presented at the 18th International Congress of Parkinson's Disease and Movement Disorders. These trials lacked detailed data on all scales.

### Implication for further studies

The findings from this meta-analysis using mGluR5 antagonists showed a greater treatment effect on the mAIMS scores but no significant improvements on LFADLDS and UPDRS Part IV scores. Therefore, more rigorously designed RCTs with longer follow-up periods are necessary in future. A number of adverse events occurred more frequently with mGluR5 antagonist treatments than with placebo, and they were mainly central nervous system symptoms. Therefore, the balance between the efficacy and side effects requires future studies. In general, there were no reports of life-threatening events in these trials. In addition, optimal dosages of compounds need to be refined to define the therapeutic window between efficacy and unacceptable side effects in future. Furthermore, combination studies of mGluR5 antagonists with other non-glutamatergic antidyskinetic drugs would be interesting. Finally, once the efficacy of mGluR5 antagonists is established, new lines of research should focus on establishing predictors of the intervention outcomes. These predictors could include age of disease onset, types of mGluR5 antagonists and treatment regimens, and genetic influences on the response to mGluR5 antagonists. All these issues should be researched by future RCTs.

## Conclusion

In summary, mGluR5 antagonists had a greater treatment effect on mAIMS scores in LID patients without reducing the efficacy of antiparkinsonian therapy. However, there were no improvements in antidyskinesia on the LFADLDS and UPDRS Part IV scales compared with placebo. According to these results, we are unable to recommend mGluR5 antagonists for the routine treatment of LID patients right now. We need more RCTs in this field to guide the clinic.

## Author contributions

W-WW, X-RZ, and Z-RZ conceived and participated in its design, searched databases, S-YC extracted and assessed studies and helped to draft the manuscript. JC helped in guiding and revising the manuscript. X-SW and C-LX participated in the conceptualization and design of the review and revised the review. All authors read and approved the final manuscript.

### Conflict of interest statement

The authors declare that the research was conducted in the absence of any commercial or financial relationships that could be construed as a potential conflict of interest.

## References

[B1] Abdel-Daim M. Funasaka Y. Komoto M. Nakagawa Y. Yanagita E. Nishigori C. (2010). Pharmacogenomics of metabotropic glutamate receptor subtype 1 and *in vivo* malignant melanoma formation. J. Dermatol. 37, 635–646. 10.1111/j.1346-8138.2010.00833.x 20629830

[B2] Atkins D. Best D. Briss P. A. Eccles M. Falck-Ytter Y. Flottorp S. (2004). GRADE Working Group.Grading quality of evidence and strength of recommendations. BMJ 328:1490 10.1136/bmj.328.7454.1490 15205295 PMC428525

[B3] Bastide M. F. Meissner W. G. Picconi B. Fasano S. Fernagut P. O. Feyder M.. (2015). Pathophysiology of L-dopa-induced motor and non-motor complications in Parkinson's disease. Prog. Neurobiol. 132, 96–168. 10.1016/j.pneurobio.2015.07.002 26209473

[B4] Berg D. Godau J. Trenkwalder C. Eggert K. Csoti I. Storch A.. (2011). AFQ056 treatment of levodopa-induced dyskinesias: results of 2 randomized controlled trials. Mov. Disord. 26, 1243–1250. 10.1002/mds.23616 21484867

[B5] Bibbiani F. Oh J. D. Kielaite A. Collins M. A. Smith C. Chase T. N. (2005). Combined blockade of AMPA and NMDA glutamate receptors reduces levodopa-induced motor complications in animal models of PD. Exp. Neurol. 196, 422–429. 10.1016/j.expneurol.2005.08.017 16203001

[B6] Bonsi P. Cuomo D. De Persis C. Centonze D. Bernardi G. Calabresi P.. (2005). Modulatory action of metabotropic glutamate receptor (mGluR) 5 on mGluR1 function in striatal cholinergic interneurons. Neuropharmacology 49(Suppl. 1), 104–113. 10.1016/j.neuropharm.2005.05.012 16005029

[B7] Calabresi P. Di Filippo M. Ghiglieri V. Tambasco N. Picconi B. (2010). Levodopa-induced dyskinesias in patients with Parkinson's disease: filling the bench-to-bedside gap. Lancet Neurol. 9, 1106–1117. 10.1016/S1474-4422(10)70218-0 20880751

[B8] Chase T. N. Oh J. D. (2000). Striatal dopamine- and glutamate-mediated dysregulation inexperimental parkinsonism. Trends Neurosci. 23(10 Suppl.), S86–91. 10.1016/S1471-1931(00)00018-5 11052225

[B9] Chou Y. H. Hickey P. T. Sundman M. Song A. W. Chen N. K. (2015). Effects of repetitive transcranial magnetic stimulation on motor symptoms in Parkinson disease: a systematic review and meta-analysis. JAMA Neurol. 72, 432–440. 10.1001/jamaneurol.2014.4380 25686212 PMC4425190

[B10] Del-Bel E. Bortolanza M. Dos-Santos-Pereira M. Bariotto K. Raisman-Vozari R. (2016). l-DOPA-induced dyskinesia in Parkinson's disease: are neuroinflammation and astrocytes key elements? Synapse 70, 479–500. 10.1002/syn.21941 27618286

[B11] Fox S. H. Katzenschlager R. Lim S. Y. Ravina B. Seppi K. Coelho M. (2011). The Movement disorder society evidence-based medicine review update: treatments for the motor symptoms of Parkinson's disease. Mov. Disord. 26(Suppl. 3), S2–41. 10.1002/mds.23829 22021173

[B12] Goetz C. G. Stebbins G. T. Chung K. A. Hauser R. A. Miyasaki J. M. Nicholas A. P.. (2013). Which dyskinesia scale best detects treatment response? Mov. Disord. 28, 341–346. 10.1002/mds.25321 23390076

[B13] Higgins J. P. Altman D. G. Gøtzsche P. C. Jüni P. Moher D. Oxman A. D. (2011). Cochrane bias methods group; cochrane statistical methods group. the cochrane collaboration's tool for assessing risk of bias in randomised trials. BMJ 343:d5928 10.1136/bmj.d5928 22008217 PMC3196245

[B14] Higgins J. P. Thompson S. G. Deeks J. J. Altman D. G. (2003). Measuring inconsistency in meta-analyses. BMJ 327, 557–560. 10.1136/bmj.327.7414.557 12958120 PMC192859

[B15] Jenner P. (2008). Molecular mechanisms of L-DOPA-induced dyskinesia. Nat. Rev. Neurosci. 9, 665–677. 10.1038/nrn2471 18714325

[B16] Kumar R. Hauser R. A. Mostillo J. Dronamraju N. Graf A. Merschhemke M.. (2016). Mavoglurant (AFQ056) in combination with increased levodopa dosages in Parkinson's disease patients. Int. J. Neurosci. 126, 20–24. 10.3109/00207454.2013.841685 24007304

[B17] Levandis G. Bazzini E. Armentero M. T. Nappi G. Blandini F. (2008). Systemic administration of an mGluR5 antagonist, but not unilateral subthalamic lesion,counteracts l-DOPA-induced dyskinesias in a rodent model of Parkinson's disease. Neurobiol. Dis. 29, 161–168. 10.1016/j.nbd.2007.08.011 17933546

[B18] Marconi R. Lefebvre-Caparros D. Bonnet A. M. Vidailhet M. Dubois B. Agid Y. (1994). Levodopa-induced dyskinesias in Parkinson's disease phenomenology and pathophysiology. Mov. Disord. 9, 2–12. 10.1002/mds.870090103 8139601

[B19] Morin N. Grégoire L. Gomez-Mancilla B. Gasparini F. Di Paolo T. (2010). Effect of the metabotropic glutamate receptor type 5 antagonists MPEP and MTEP in parkinsonian monkeys. Neuropharmacology 58, 981–986. 10.1016/j.neuropharm.2009.12.024 20074579

[B20] Ohtani Y. Harada T. Funasaka Y. Nakao K. Takahara C. Abdel-Daim M.. (2008). Metabotropic glutamate receptor subtype-1 is essential for *in vivo* growth of melanoma. Oncogene 27, 7162–7170. 10.1038/onc.2008.329 18776920

[B21] Ouattara B. Grégoire L. Morissette M. Gasparini F. Vranesic I. Bilbe G.. (2011). Metabotropic glutamate receptor type 5 in levodopa-induced motor complications. Neurobiol. Aging 32, 1286–1295. 10.1016/j.neurobiolaging.2009.07.014 20036444

[B22] Parkinson Study Group (2001). Evaluation of dyskinesias in a pilot, randomized,placebo-controlled trial of remacemide in advanced Parkinson disease. Arch. Neurol. 58, 1660–1668. 10.1001/archneur.58.10.1660 11594926

[B23] Reiner A. Levitz J. (2018). Glutamatergic signaling in the central nervous system: ionotropic and metabotropic receptors in concert. Neuron 98, 1080–1098. 10.1016/j.neuron.2018.05.018 29953871 PMC6484838

[B24] Samadi P. Grégoire L. Morissette M. Calon F. Hadj Tahar A. Bélanger N.. (2008). Basal ganglia group II metabotropic glutamate receptors specific binding in non-human primate model of L-Dopa-induced dyskinesias. Neuropharmacology 54, 258–268. 10.1016/j.neuropharm.2007.08.009 18001807

[B25] Stocchi F. Balaguer E. Trenkwalder C. Shah A. Dronamraju N. Tran M. (2014). 12-Week, Double-Blind, Placebo-Controlled, Fixed-Dose Study of Immediate Release AFQ056, an mGluR5 Receptor Antagonist, in Parkinson's Disease Patients with Moderate-to-Severe l-Dopa Induced Dyskinesias. Stockholm: International Congress of Parkinson's Disease and Movement Disorders.

[B26] Stocchi F. Rascol O. Destee A. Hattori N. Hauser R. A. Lang A. E.. (2013). AFQ056 in Parkinson patients with levodopa-induced dyskinesia: 13-week, randomized, dose-finding study. Mov. Disord. 28, 1838–1846. 10.1002/mds.25561 23853029

[B27] Tison F. Keywood C. Wakefield M. Durif F. Corvol J. C. Eggert K.. (2016). A phase 2A trial of the novel mGluR5-negative allosteric modulator dipraglurant for levodopa-induced dyskinesia in Parkinson's disease. Mov. Disord. 31, 1373–1380. 10.1002/mds.26659 27214664

[B28] Trenkwalder C. Kulisevsky J. Poewe W. Shah A. Han G. Tran M. (2014). 13-Week, Double-Blind, Placebo-Controlled, Fixed-Dose Study of Modified Release AFQ056, an mGluR5 Receptor Antagonist, in Parkinson's Disease Patients With Moderate-to-Severe L-Dopa Induced Dyskinesias. Stockholm: International Congress of Parkinson's Disease and Movement Disorders.

[B29] Trenkwalder C. Stocchi F. Poewe W. Dronamraju N. Kenney C. Shah A.. (2016). Mavoglurant in Parkinson's patients with l-Dopa-induced dyskinesias: two randomized phase 2 studies. Mov. Disord. 31, 1054–1058. 10.1002/mds.26585 27214258

[B30] Vesterinen H. M. Sena E. S. Egan K. J. Hirst T. C. Churolov L. Currie G. L.. (2014). Meta-analysis of data from animal studies: a practical guide. J. Neurosci. Methods 221, 92–102. 10.1016/j.jneumeth.2013.09.010 24099992

[B31] Xie C. L. Shao B. Chen J. Zhou Y. Lin S. Y. Wang W. W. (2016). Effects of neurostimulation for advanced Parkinson's disease patients on motor symptoms: a multiple-treatments meta-analysas of randomized controlled trials. Sci. Rep. 6:25285. 10.1038/srep25285 27142183 PMC4855136

